# Metachronous pulmonary metastasis after radical esophagectomy for esophageal cancer: prognosis and outcome

**DOI:** 10.1186/1749-8090-7-103

**Published:** 2012-10-02

**Authors:** Masashi Takemura, Katsunobu Sakurai, Mamiko Takii, Kayo Yoshida

**Affiliations:** 1 Department of Upper Gastrointestinal Surgery, Hyogo College of Medicine, 1-1, Mucogawa-machi, Nishinomiya City, Hyogo 663-8501, Japan; 2 Department of Gastrointestinal Surgery, Osaka City General Hospital, 1-8-20, Nagasu Nishidori, Amagasaki City, Hyogo, 660-0807, Japan

**Keywords:** Metachronous pulmonary metastasis, Esophageal cancer, Metastasectomy

## Abstract

**Background:**

Few reports discuss the outcome of pulmonary metastasis after radical esophagectomy for esophageal cancer. To clarify the data from such cases, we conducted a retrospective study on the clinical outcome of patients who developed pulmonary metastasis after undergoing radical esophagectomy.

**Methods:**

We retrospectively reviewed the prognosis and clinical outcome of 25 patients who developed metachronous pulmonary metastasis after esophagectomy for esophageal cancer.

**Results:**

The site of recurrence was pulmonary without extrapulmonary metastasis in 14 patients and extrapulmonary metastasis was observed in 11. Nineteen patients had multiple pulmonary metastasis and 6 had solitary pulmonary metastasis. Twenty-four of patients underwent systemic chemotherapy during initial treatment for metastatic lesions. Pulmonary metastasectomy was indicated in 5 patients with solitary metastasis. The actual 1-, 2- and 4-year survival rates were 60%, 36% and 27%, respectively. Gender, operative procedure, and postoperative morbidity were not significant prognostic factors. However, pathological staging of primary esophageal cancer was a significant prognostic factor. Survival was significantly worse in patients who did not undergo resection than in those who did. The number of pulmonary metastasis, complicated extrapulmonary metastasis and the time of recurrence were also significant prognostic factors.

**Conclusions:**

Multiple pulmonary metastases or complicated extrapulmonary metastasis were unfavorable prognostic factors for patients with pulmonary metastasis arising from esophageal cancer. Although, surgical intervention is not recommended in such cases, metastasectomy is an acceptable choice of treatment for solitary pulmonary metastasis.

## Background

Esophageal cancer is a highly lethal disease, and one of the most common neoplasms. Recent advances in multimodal treatment, including esophagectomy with three-field lymph node dissection and definitive chemoradiotherapy, have improved the prognosis of patients with esophageal cancer [1-3]. Particularly, patient survival after esophagectomy with three-field lymph node dissection is better than that after the two-field procedure [4]. However, the disease recurs after radical esophagectomy in 30%-50% patients with hematogenous recurrence accounting for 50% of these cases [5-7].

Hematogenous metastases are commonly found in the liver, bones and lungs [6,7]. However, few reports discuss the outcome of pulmonary metastasis after radical esophagectomy for esophageal cancer [8,9]. To clarify the data from such cases, we conducted a retrospective study on the clinical outcome of patients who developed pulmonary metastasis after undergoing radical esophagectomy at our department.

## Methods

We retrospectively analyzed patients who developed metachronous pulmonary metastasis after undergoing esophagectomy for esophageal cancer at Osaka City General Hospital, Osaka Japan between January 2000 and March 2010. During this period, 373 patients with esophageal cancer have been underwent radical esophagectomy with reconstruction. All patients underwent complete radical resection for primary esophageal cancer. After surgery, patients were monitored on an outpatient basis at 3-month intervals for 2 years, and at 6-month intervals thereafter ( Additional file 1: Table S1). No patients died during the initial hospital stay or within 1 month of surgery after surgery. Of these 373 patients, 25 (6.7%) were diagnosed with metachronous pulmonary metastasis after esophagectomy. Recurrent disease was frequently diagnosed by chest X-rays, tumor markers, or computed tomography and occasionally by positron emission tomography. Further examinations were also performed to diagnose extrapulmonary metastases. The last follow-up data for this study population was gathered in January 2011, and the median follow-up duration was 29.2 months (range: 5–124 months).

The therapeutic approach to each metastasis was selected by individual surgeons. This was solely done on the basis of their knowledge and experience, and not any preset common criteria, institutional protocols or guidelines. Treatment options for pulmonary metastasis included best supportive care, systemic chemotherapy, and pulmonary metastasectomy. The selection criteria of pulmonary metastatectomy from esophageal carcinoma were as follows; 1) the patient has a performance status (PS) of 0 or 1 based on ECOG scale, 2) patient can tolerate surgical intervention, 3) the metastasic lesion was diagnosed as solitary metastasis without pleural dissemination, 4) there are no metastatic lesions other than lung lesion. Resected specimens were referred to the department of pathology in our department by at least 2 experienced pathologists. A pulmonary lesion was diagnosed as a metastasis from esophageal cancer on histological findings as follows; a similarly to the primary esophageal tumor; a tumor progression originating from the bronchial subepithelium; and/or a discontinuity between the tumor and bronchial epithelium.

We gathered the following perioperative demographic and clinical data from patient records: age, gender, chest / abdominal surgical procedure, type of recurrence, and treatment following diagnosis with pulmonary metastasis. Furthermore, we evaluated the depth of invasion, extent of lymph node metastases, lymphatic and venous invasion, and the pathological diagnosis and classification of primary esophageal cancer using the TNM classification given by the Union for International Cancer Control [10] and the Japanese classification of esophageal cancer given by the Japanese Society for Esophageal Diseases [11].

The following clinicopathological factors were analyzed for prognostic significance using the Kaplan-Meier method and log-rank test; age, gender, initial surgical procedure, location of esophageal tumor, pattern of recurrence, tumor classification, depth of tumor invasion, extent of lymph node metastasis, pathological staging, lymphatic and venous invasion of the primary tumor, number of pulmonary metastatic lesions, time of recurrence, and presence or absence of pulmonary resection for metastatic lesions. P < 0.05 was considered statistically significant. Statistical analysis was performed using statistical analysis program package (SPSS 11.0; SPSS Inc., Chicago, Illinois, USA). This study was conducted in accordance with institutional guidelines of the Ethics Committee for Clinical Rresearch of Osaka City General Hospital.

## Results

The clinical characteristics of patients are summarized in Additional file 2: Table S2. There were 21 men and 4 women with a mean age of 65.7 years (range 48–75) at the time of esophagectomy. Twenty-one patients underwent thoracoscopic esophagectomy, and 4 underwent esophagectomy via thoracotomy. An abdominal approach using laparoscopy was adopted in 19 patients and a gastric roll was used as an esophageal substitute in 22 patients. All patients underwent complete resection for the primary esophageal lesion (R0). Pathological TNM stages of the primary esophageal cancer were as follows; stage IA in 4 patients (17%); IIA in 3 (13%); IIB in 2 (8%); IIIA in 6 (24%); IIIB in 6 (24%); and IIIC in 4 (17%). Tumor classifications included as follows; squamous cell carcinoma in 20 patients, adenocarcinoma in 4, and basaloid squamous cell carcinoma in 1. Seventeen patients (68%) exhibited lymph node metastasis. Lymphatic invasion was diagnosed in 10 patients, and venous was diagnosed in 4. Time of recurrence after initial surgery was as follows; ≦2 years in 16 patients (64%) and > 2 years in 9 (36%). Site of recurrence was only pulmonary metastases in 14 patients, and extrapulmonary metastasis was observed in 11 patients (lymph node metastasis in 3, other hematogenous metastases in 7, and complicated metastasis in 1). Nineteen patients had multiple pulmonary metastases and 6 had solitary pulmonary metastasis. Twenty-four of patients underwent systemic chemotherapy during initial treatment for metastatic lesions. Pulmonary metastasectomy was indicated in 5 patients with solitary metastasis; the clinicopathological data of primary esophageal cancer in these 5 patients are summarized in Table 1. Three patients were diagnosed with upper thoracic esophageal cancer and 2 with lower thoracic esophageal cancer. Lymph node metastasis was histologically diagnosed in 2 patients, while lymphatic invasion was positive in 1. The tumor invaded the adventitia in 2 patients, and was submucosal in 3. All 5 patients with metastasectomy had a good performance status and underwent combined chemotherapy with 5-fluorouracil and cisplatin before metastasectomy (Table 2). All patients underwent wedge resection. None died as a result of metastasectomy, and all were discharged without recurrent disease. Only 1 patient received chemotherapy after metastasectomy, this patient died of multiple metastatic tumors 6 months after metastasectomy. Four patients are currently alive, without any evidence of metastatic disease, 124, 65, 48 and 32 months after esophagectomy, respectively.

**Table 1:** Clinicopathological data on initial esophageal cancer in metastasectomy patients

**Case**	**Gender**	**Age**	**Tumor**	**location**	**pT**	**pN**	**1y**	**v**	**Pathology**
1	M	57	Upper	thoracic	pT1b	pN0	0	0	SCC
2	M	58	Upper	thoracic	pT3	pN0	0	0	SCC
3	M	74	Lower	thoracic	pT3	pN1	+	0	SCC
4	F	63	Lower	thoracic	pT1b	pN1	0	0	Basaloid
5	M	75	Upper	thoracic	pT1b	pN0	0	0	SCC

ly: lumphatic invasion.

v: venous invasion.

PS: ECOG performance status.

SCC: Squamous cell carcimona.

**Table 2:** Clinical data on pulmonary metastases in metastasectomy patients

**Case**	**Gender**	**PS**	**CT pre/post metastasectomy**	**Operative procedure**	**OS**	**Survival**
1	M	0	Yes/No	Wedge resection	1305	Dead
2	M	0	Yes/No	Wedge resection	1938	Alive
3	M	0	Yes/No	Wedge resection	2599	Alive
4	F	0	Yes/No	Wedge resection	4024	Alive
5	M	0	Yes/No	Wedge resection	1260	Alive

CT: chemotherapy.

Patient survival according to prognostic factors is shown in Table 3. The actual 1-, 2- and 4-year survival rates were 60%, 36% and 27%, respectively. Gender, operative procedure, and postoperative morbidity were not significant prognostic factors. However, pathological staging of the primary esophageal cancer was a significant prognostic factor. The actual 1- and 4-year survival rates were 66.7% and 53.5% for stage I or II, respectively, and 56.3% and 12.5% for stage III or IV, respectively. Lymphatic and venous invasion were not significant factors. Survival was significantly worse in patients who did not undergo resection than in those who did. Number of pulmonary metastasis lesions (solitary versus multiple), extrapulmonary metastasis and time of recurrence were also significant prognostic factors.

**Table 3:** Survival rate according to clinicopathological factors

**Factors related survival**		**n**	**survival rate**			
			**1 year**	**2 year**	**4 year**	**p value**
All cases		25	60.0	36.0	27.5	
Gender	Male	21	57.1	28.6	22.9	0.424
	Female	4	75.0	75.0	50.0	
Thoracic procedure	Thoracotomy	21	75.0	50.0	50.0	0.402
	Thoracoscopy	4	57.1	33.3	22.2	
Postoperative morbidity	Present	11	72.7	45.5	36.3	0.162
	Absent	14	50.0	28.6	21.4	
pT	pT1	8	75.0	50.0	33.3	0.562
	pT3, pT4	17	52.9	29.4	23.5	
pN	+	18	61.1	27.8	22.2	0.707
	-	7	57.1	57.1	38.1	
pStage	I, II	9	66.7	66.7	53.3	0.042
	III, IV	16	56.3	18.8	12.5	
Classification of the tumor	Squamous cell csrcinoma	20	50.0	25.0	18.8	0.052
	Other type	5	80.0	80.0	60.0	
Lymphatic invasion	+	10	44.4	11.1	11.1	0.218
	-	15	68.8	50.0	36.4	
Venous invasion	+	4	50.0	0	0	0.290
	-	21	61.9	42.9	32.1	
Number of pulmonary metastasis	Solitary	6	100.0	100.0	80.0	0.001
	Multiple	19	47.4	15.8	10.5	
Extrapulmonary metastasis	Present	11	45.8	18.2	0	0.016
	Absent	14	71.4	50.0	41.7	
Pulmonary resection	Resected	5	100.0	100.0	75.0	0.002
	Nonresected	20	50.0	20.0	15.0	
Time of recurrence	Within 2 year	16	37.5	0.0	0.0	0.001
	More than 2 years	9	100.0	100.0	76.2	

## Discussion

In Japan, radical esophagectomy with three-field lymphadenectomy is recommended as the first line of treatment for locally advanced esophageal cancer [1,2,4]. However, the recurrence rate of esophageal cancer is high even after radical esophagectomy [1,2,4,5]. Nakagawa *et al*. [7] reported an overall 5-year survival rate of 55.6% after three-field lymph node dissection. In this study, recurrence was most frequently locoregional followed by after hematogenous recurrence. Kunisaki *et al.*[6] also reviewed patients with tumor recurrence after curative esophagectomy, and reported that the tumors recurred in 43.4% patients, and almost all cases recurred within 2 years after surgery (88.9%). Moreover, the survival of patients with distant metastasis was significantly worse than that of patients with locoregional recurrence. In these reports, the prognosis of the patients with hematogenous recurrence was poor despite aggressive multimodal treatment.

Law *et al.*[5] reported on the site of recurrence in 56 patients who underwent curative resection. Recurrence was most frequent in the lungs (13/56), followed by the liver (11/56). Mafune *et al.*[10] reviewed autopsy findings in 69 patients with esophageal cancer (33 of whom had underwent resection, whereas 36 did not). The liver and lungs were the most common sites of hematogenous metastasis. A high incidence of hematogenous metastasis suggests that stronger chemotherapy is required, irrespective of resection. In our study, 24 patients were treated with systemic chemotherapy for pulmonary metastasis; the disease persisted in only 1 patient after chemotherapy. It is important to develop effective chemotherapeutic regimens to improve the outcome and prognosis of such cases. We previously reported the detection of 5-fluorouracil-related enzymes in esophageal cancer tissue [11], thus supporting the use of this chemotherapeutic agent against esophageal cancer.

To date, few reports have discussed the surgical treatment of pulmonary metastasis arising from esophageal cancer [8,9,12]. As hematogenous recurrence of esophageal cancer is considered a systemic disease, surgical treatment is rarely indicated. Ichikawa *et al.*[9] reviewed 23 patients who underwent surgical treatment for metachronous pulmonary metastasis arising from esophageal cancer. They concluded that surgical intervention for metachronous pulmonary metastasis is an acceptable treatment option in select cases without extrapulmonary metastasis. In our patients, the 1- and 2-year survival rates of patients who did not undergo resection were 50% and 20%, respectively; these rates were significantly worse those of patients who underwent resection. Median survival time from esophagectomy was 366.5 days for patients who did not undergo resection; conversely, 75% patients who undergo resection survived for 2 years. As the number of cases who underwent resection was limited, no definite conclusions could be drawn from our study results. However, our case series suggests that surgical intervention for metastatic lesions arising from esophageal cancer is indicated when metastases are technically resectable and patients have a good performance status.

## Conclusion

In conclusions, multiple pulmonary metastases or complicated extrapulmonary metastasis were unfavorable prognostic factors for patients with pulmonary metastasis from esophageal cancer. Although, surgical intervention is not recommended in such cases, metastasectomy for solitary pulmonary metastasis is an acceptable treatment choice. To overcome the limitations of a small case series, further studies of patients with pulmonary metastasis arising from esophageal cancer are necessary to determine the selection criteria for surgical intervention.

## Competing interests

The authors declare that they have no competing interests.

## Authors’ contributions

All authors participated in the acquisition of data and revision of the manuscript. AA authors determined the design, performed the statistical analysis and drafted the manuscript. All authors read and gave final approval for the version submitted for publication.

## Supplementary Material

Additional file 1 Table S1. Outpatient follow-up program after esophagectomy for esophageal cancer. Click here for file

Additional file 2 Table S2. Patient characteristics. Click here for file
