# Supplementary figures and images for: Metachronous pulmonary metastasis after radical esophagectomy for esophageal cancer: prognosis and outcome
Source: J Cardiothorac Surg. 2012 Oct 2;7:103. doi: 10.1186/1749-8090-7-103 (PMC3504510; doi:10.1186/1749-8090-7-103)

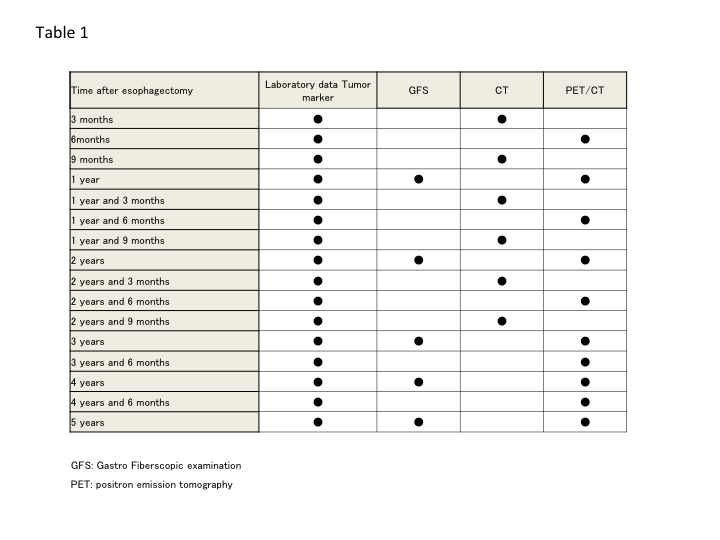

Supplement: Additional file 1 — Table S1. Outpatient follow-up program after esophagectomy for esophageal cancer. [file 1749-8090-7-103-S1.tiff]
