# Supplementary material for: Metachronous pulmonary metastasis after radical esophagectomy for esophageal cancer: prognosis and outcome
Source: J Cardiothorac Surg. 2012 Oct 2;7:103. doi: 10.1186/1749-8090-7-103 (PMC3504510; doi:10.1186/1749-8090-7-103)
Supplement: Additional file 2 — Table S2. Patient characteristics. [file 1749-8090-7-103-S2.pdf]

		All cases (n=25)
Age	(year)	65.7 ± 6.9
Gender	Male	21
	Female	4
Location of tumor	Upper thoracic esophagus	4
	Middle thoracic esophagus	13
	Lower thoracic esophagus	8
Thoracic approach	Thoracoscopy	21
	Thoracotomy	4
Abdominal approach	Laparpscopy	19
	Laparotomy	6
Reconstruction organ	Stomach	22
	Pedicled jejunum	3
Postoperative morbidity	Present	11
	Absent	14
Classification of main tumor	Squamous cell carcinoma	20
	Adenocarcinoma	4
	Basaloid	1
pT(UICC)	pT1a	1
	pT1b	7
	pT2	0
	pT3	16
	pT4	1
pN(UICC)	pN0	8
	pN1	5
	pN2	8
	pN3	4
pStage (UICC)	IA	4
	IIA	3
	IIB	2
	IIIA	6
	IIIB	6
	IIIC	4
Lymphatic invasion	+	10
	-	15
Venous invasion	+	4
	-	21
Pulmonary metastasis	Solitary	6
	Multiple	19
Extrapulmonary metastasis	Present	11
	Absent	14
Chemotherapy for pulmonary metastasis	Present	24
	Absent	1
Pulmonary resection	Resected	5
	Nonresected	20
Time of recurrence (from esophagectomy)	Within 2 year	16
	More than 2 years	9
